# Occupational exposure of health care personnel to SARS-CoV-2 particles in the intensive care unit of Tehran hospital

**DOI:** 10.1007/s13762-020-03095-z

**Published:** 2021-02-02

**Authors:** R. Yarahmadi, F. Bokharaei-Salim, S. Soleimani-Alyar, P. Moridi, O. Moradi-Moghaddam, M. Niakan-Lahiji, M.-M. Darvishi, S. Golmahammadi, S. A. J. Mousavi, H. Ebrahimi, A. Ashtarinezad, A.-A. Farshad, A. Jonidi-Jafari, S. J. Kiani, S. Garshasbi, S. Mehrzadi

**Affiliations:** 1 Air Pollution Research Center, Department of Occupational Health, Iran University of Medical Sciences, Tehran, Iran; 2 Department of Virology, Faculty of Medicine, Iran University of Medical Sciences, Tehran, Iran; 3 Air Pollution Research Center, Iran University of Medical Sciences, Tehran, Iran; 4 Air Pollution Research Core, Pars Plasma Bonyan (Knowledge Based Co), Tehran, Iran; 5 Trauma and Injury Research Center, Critical Care Medicine Department, Iran University of Medical Sciences, Tehran, Iran; 6 FCCM. Anesthesiology and Critical Care Department, Trauma and Injury Research Center, Rasool-E-Akram Complex Hospital, Iran University of Medical Sciences, Tehran, Iran; 7 Department of Engineering, Payame Noor University, Tehran, Iran; 8 Air Pollution Research Center, Department of Occupational Health, Iran University of Medical Sciences, Tehran, Iran; 9 Occupational Health Research Center, Department of Occupational Health, Iran University of Medical Sciences, Tehran, Iran; 10 Department of Environmental Health, Iran University of Medical Sciences, Tehran, Iran; 11 School of Medicine, Iran University of Medical Sciences, Tehran, Iran; 12 Vice Chancellor for Health Center, Iran University of Medical Sciences, Tehran, Iran; 13 Razi Drug Research Center, Iran University of Medical Sciences, Tehran, Iran

**Keywords:** SARS-CoV-2, Airborne, Health care, RT-PCR, Impingement, COVID-19

## Abstract

The outbreak of SARS-CoV-2 (COVID-19) has attracted much attention to study its possible presence and airborne transmission. The possibility of COVID-19 airborne transmission in indoor environments is debatable. The present study examined the concentration of viral RNA-containing particles produced directly or indirectly by breathing or coughing of confirmed COVID-19 patients or by carriers without symptoms. Some studies do not accept this method of transmission (COVID-19 airborne transmission). The present study aimed to measure the possible exposure of health care personnel to SARS-CoV-2 particles that may have been suspended in the air to respond to the hypothesis of COVID-19 airborne transmission. Airborne particle sampling was performed using impingement method based on NIOSH (chapter BA) and ASHRAE. Selection of sampling sections was in line with the WHO guidelines. The samples were analyzed using RT-PCR technique. Based on the given results, airborne particles of COVID-19 may present in the air and affect the health of hospital personnel. In fact, the analysis of gene expression in ambient conditions and thereby aerosol transmission of SARS-CoV-2 through air is possible and may lead to occupational exposure of health care personnel. Furthermore, it was found that airborne emission of COVID-19 through the breathing zone of patients, particularly in ICU wards with confirmed cases of COVID-19, may be higher than in other ICU wards. Also, the demonstrated results showed that there is a possibility of reaerosolization (reintroduction) of previously airborne SARS-CoV-2 particles into the atmosphere due to health care personnel frequently walking between different wards and stations of ICU.

## Introduction

Bioaerosols are very small airborne particles (0.001 to 100 μm) that originate from plants/animals and can contain living organisms (Georgakopoulos, et al. 2009; Mandal and Brandl 2011). Pathogenic or nonpathogenic dead or alive microorganisms (e.g., viruses, bacteria, and fungi) may exist in bioaerosols (Salthammer and Uhde 2009). Sources of bioaerosol exposure in occupational activities are diverse enough to include waste sorting and composting, agricultural and food processing activities, the livestock industry, etc. (Kim, et al. 2018). Health care occupational exposure to bioaerosol can result in the deposition of the pathogen in the respiratory tract of the host causing disease and an immunological response. (Guzman 2020).

COVID-19, which was first reported in Wuhan, China, in late December 2019, became a pandemic rapidly (Qiu et al. 2020), and by June 20, 2020, more than 216 countries and territories reported a total of 8,525,042 confirmed cases and 456,973 confirmed deaths. (WHO 2020) Great efforts have been made to enhance scientific facts related to COVID-19. (Guzman 2020; Qiu et al. 2020).

COVID-19 outbreak attracted much attention to study the possible transmission ways through bioaerosols. (Guzman 2020; WHO Organization 2020b) Respiratory droplets have a size distribution range of between a submicron to thousands of microns. Droplets with larger than 100 microns stay in air less than for 5 s in height of 1.5 m from the ground. (Marr et al. 2019) Droplets larger than 10 microns settle faster by gravity (Crowe et al. 1998), but droplets less than 10 microns stay in the air and spread throughout the room. (Tellier 2006) Thus, they have enough time to evaporate into droplet nuclei, with a size of 0.74 to 2.12 microns, which are involved in the airborne transmission of diseases. (Yang et al. 2007) Many respiratory particles are very small at the moment of leaving the mouth and can stay in the air for several minutes or more before they evaporate and lose their water content. However, some of the larger particles evaporate and become smaller and can stay in the air for the same extent of time. (Nicas et al. 2005) The size of the particle droplets caused by sneezing or coughing generally ranges from 1 to 5 microns. (Wang and Du 2020) Respiratory transmission occurs through inhalation of viruses deposited in the respiratory particles and sitting at the alveolar region of the lower respiratory tract. (Spicknall et al. 2010) Considerable amounts of respiratory particles are aerosolized during a usual talk. Thus, during a face to face conversation, there is a possibility of virus transmission by a COVID-19 carrier person. (Asadi et al. 2020) Some cases of infection by COVID-19 are reported in people without any contact with confirmed cases; thus, COVID-19 transmission through aerosols is possible. (Wang and Du 2020).

The possibility of aerosolization of COVID-19 in the air and its viability on the surfaces has been evaluated recently based on the results which explained the resistance and viability of COVID-19 is similar to SARS-CoV-1. Also, the high viral load of SARS-CoV-2 in upper respiratory tract and the potential of COVID-19 carrier persons in asymptomatic state for disease transmission are highly important in the epidemiologic differences. Also, the viability of SARS-CoV-2 in the air for 3 h has been confirmed. (van Doremalen et al. 2020) However, the WHO has criticized these results. It was argued that the study (van Doremalen Bushmaker Morris Holbrook Gamble Williamson Tamin Harcourt, Thornburg and Gerber 2020) was performed under laboratory conditions and a 3-jet impactor nebulizer jet was used to produce aerosols, and then, the aerosols were injected into a Goldberg drum. Also, it was emphasized that the high power of this machine cannot reflect the normal sneezing or coughing of people. (WHO Organization 2020a) A recently published work recommended considering the hypothesis of airborne transmission of SARS-CoV-2 through air and emphasized the application of control devices. (Hadei et al. 2020) However, the problem of estimating the viral load emitted, which is fundamental for the simulation of airborne transmission, has not yet been solved. (Buonanno et al. 2020).

The present study examines the following hypothesis: The airborne transmission of SARS-CoV-2 through the presence of the virus in the air and the possibility of occupational exposure of health care providers to the virus. This study was done in the intensive care unit of (ICU) of a teaching hospital in Tehran, Iran.

## Materials and methods

### Air sampling

Bioaerosol samples are usually collected into liquid media or on solid filters for measuring the particles containing virus. (Brosseau et al. 1994; Lindsley, et al. 2017; McDermott 2004; Verreault, et al. 2008) As they do not dry out and keep their viability, the use of liquid media may cause less stress on the bioaerosol components. (Lehtinen et al. 2013) Nonetheless, as such an application is still limited for analysis of the effects of size segregation, it is less preferable to conduct in-depth assessment of bioaerosols. Airborne particles are collected into a liquid collection medium in impingers method. Impingers are operated by channeling air flow through nozzles to the collection chamber containing liquid. Hence, a number of factors (eg*,* the air flow rate, distance between nozzle outlet and the surface of the liquid) influence the size diameter of the particles to be collected. (Han and Mainelis 2012).

The sampling efficiency of mentioned samplers depends on the many environmental and methodological factors influencing the integrity of the virus structure. Aerosol aerodynamic size has a direct impact on the collection efficiency of sampling device. (Verreault Moineau and Duchaine 2008).

Based on the literature reviews, impingement method was selected for bioaerosol sampling. This method was introduced by NIOSH (capture BA) and the American Society of Heating, Refrigerating and Air Conditioning Engineers (ASHRAE) as one of the bioaerosol sampling techniques. (Brosseau, Vesley, Chen, Gabel, Kuehn and Goyal 1994; Faridi et al. 2020; Girlando 2014; Lindsley, Green, Blachere, Martin, Law, Jensen and Schafer 2017; McDermott 2004) The sampling train consisted of a vacuum SKC personal pump supplying 2.5 Lit/min (Brosseau, Vesley, Chen, Gabel, Kuehn and Goyal 1994; McDermott 2004), which was calibrated with a standard flow meter before sampling and then connected to a midget impinger by Tygon tubes. The midget impinger was filled with 10 mL of HBSS. (Girlando 2014).

**Fig. 1:**
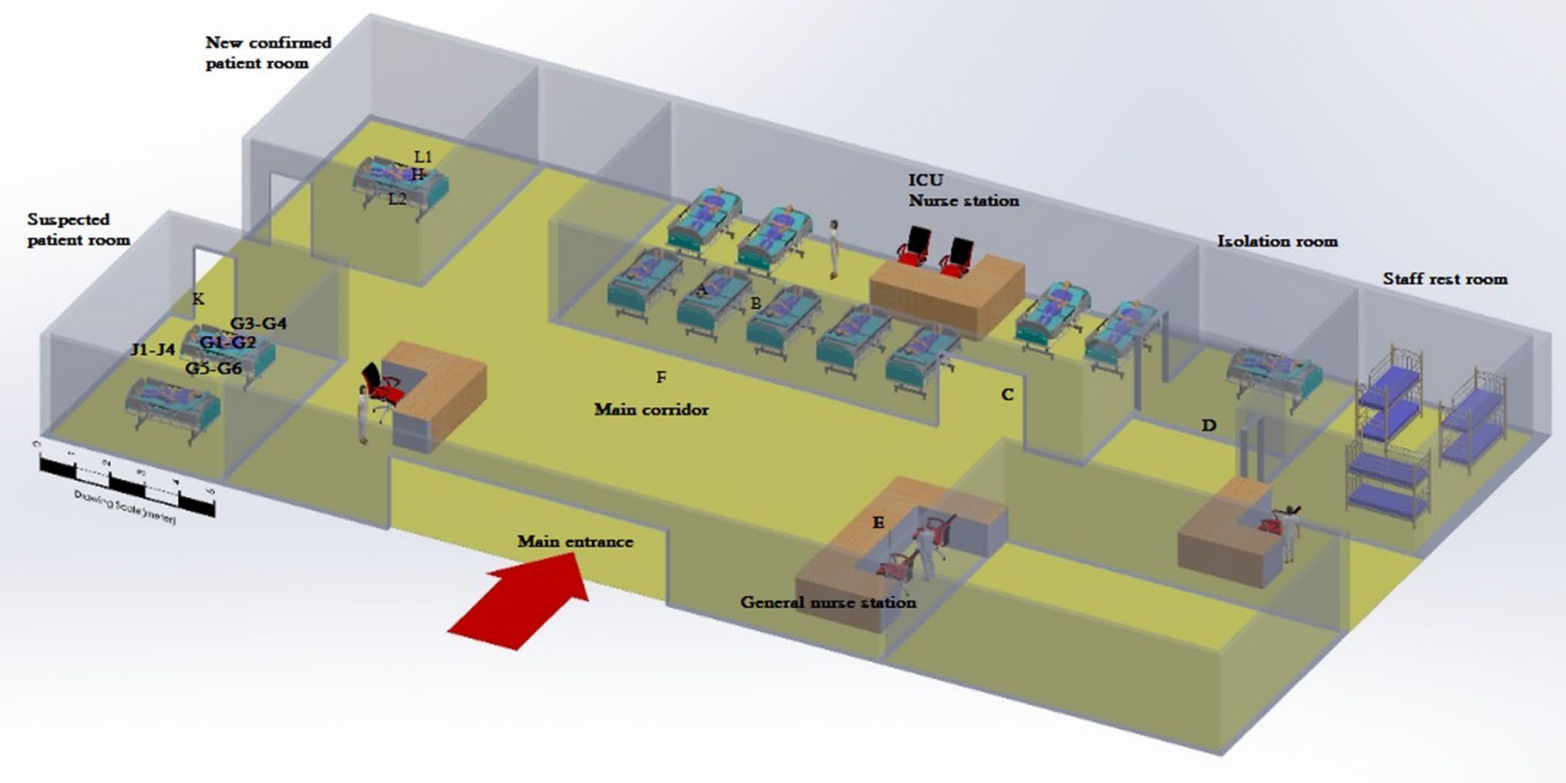
The location of sampling points (English alphabet A-L) and COVID-19 Patient’s beds (blue), nurse station, and rest room

Sampling was done at ambient temperature (24 °C) and pressure (0.88 bar), with 34 relative humidity (% RH). The sampling time for each sample was about 20 min, which accounted for the total sample volume of 50 litters. After sampling, the midget openings impinge were caped and immediately sent to the laboratory. (Brosseau, Vesley, Chen, Gabel, Kuehn and Goyal 1994; McDermott 2004) The selected sampling stations are given in Fig. 1. At the sampling time, the central air conditioner system was intentionally turned off.

The sampling locations were selected to assist in evaluation of the hypotheses about possible health care exposures based on the WHO and American Society for Testing and Materials (ASTM 2014a) (ASTM 2014; Lindsley, Green, Blachere, Martin, Law, Jensen and Schafer 2017) recommendations for surface or air sampling. (WHO Organization 2020b) However, WHO guidelines were applied for sampling to determine the aerosol concentration containing of COVID-19 at indoor areas. Since the aim of this study was to determine occupational exposure of health care personnel, the sampling area and building characteristics of the study field were categorized (Table 1) based on the source sampling as emission points of virus release (breathing zone of patients) (stations code: A, H, G1, G2). Also, general areas were classified as the path of people’s exposure (stations code: C, D, F, K, J1, J2, J3, J4) and the breathing zone of health care personnel as the receptors of the virus (stations code: B, E, L1, L2, G3, G4, G5, G6). Thus, a total of 20 samples were collected from air to survey the possible occupational exposure of health care personnel in the ICU wards (Table 2).

**Table 1:** Personnel and building characteristics of the study field

ICU wards	# of persons	Personnel status	Average age (Year)	Average resistance at ward (day/week)	Surface of area (m2)	Open surface^a^ (Door-Win) m2	Air-conditioning ^b^(Off/On)
COVID-19 patient hall	13	Oxygen mask: 5,Intubated: 4PPE:4	56	5	70	6	off
General site	3	PPE	28	3	40	20	Off/with natural ventilation
COVID-19 suspected patient room	3	Oxygen mask	58	1	24	6	off
Confirmed patient room	3	PPE	30	0	24	6	off
Total	22	–	158	38	–		

^a^All doors and windows were closed during sampling

^b^Air-conditioning systems off

**Table 2:** Sampling and clinical characteristics of persons at risk of exposure to SARS-COV-19

ICU wards	Station			Chest CT scan results	Clinical symptoms
	location	Codes	Sampling area (points)		
COVID-19 patient hall	Patient room	A	Source^1^	Positive	fever, sneezing, coughing
		B	Receiver^2^	Negative	**–**
		C	General^3^	Negative	**–**
	Staff rest room	D	General	Negative	**–**
General site	Nurse station	E	Receiver	Negative	**–**
	Local corridor	F	General	Negative	**–**
COVID-19 confirmed patient room	Patients breathing zone	H	Source	Positive	**–**
	Breathing zone of health care personnel	L1-2	Receiver	Negative	**–**
COVID-19 suspected patient room	Room entrance	K	General	–	**–**
	General area	J1-J4	General	Negative	**–**
	Patients breathing zone	G1-G2	Source	Positive	fever, sneezing, coughing
	Breathing zone of Health care personnel	G3-G6	Receiver	Negative	**–**

**Fig. 2:**
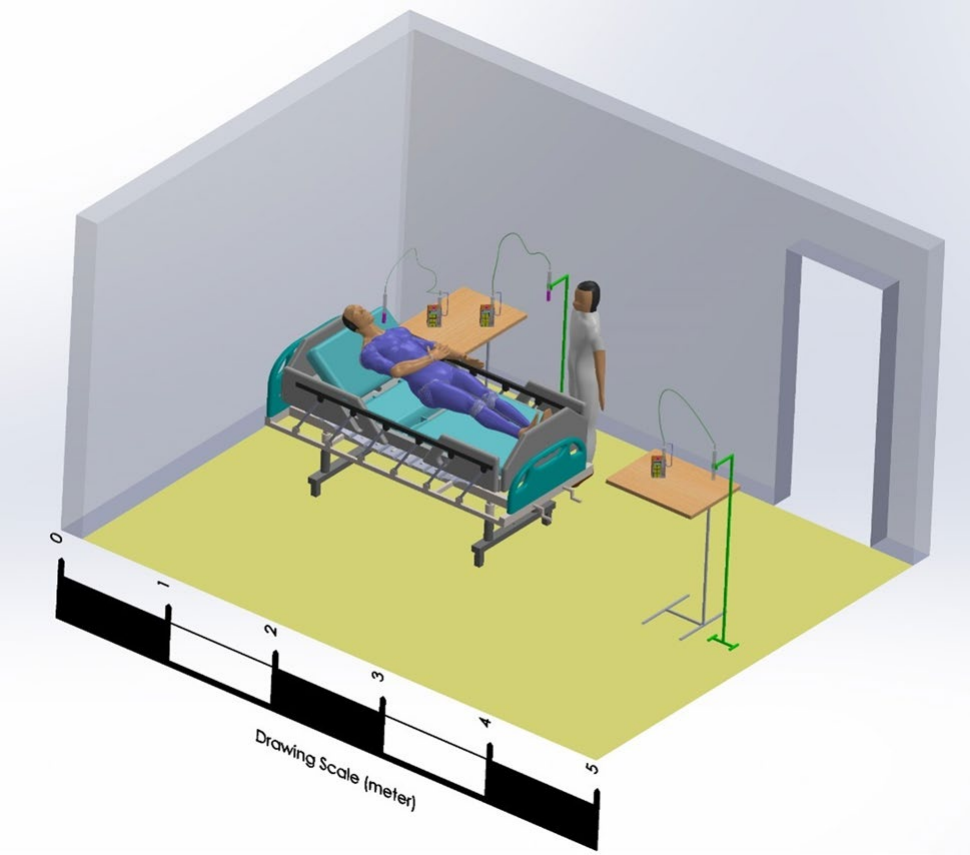
The schematic of the air sampling experiment setup in the COVID-19 ICU wards

Choosing the number and location of sampling points according to the variability, analytical methods used, the variability of contaminant concentration over time at the site, and the level of precision was required. (Figs. 1 & 2) In addition, determining the number of locations and placement of samplers was based on the nature of the response, ground level, metrological conditions, site location (according to conflicting background sources), site space, the number and size and relative proximity of other sources on the site and the above-mentioned resources. (Keith et al. 1991).

### Liquid media of sampling

The type of liquid media in bioaerosol sampling is important, as improper media may harm the virus RNA. (Girlando 2014) sVirus death during sampling may result from osmotic shock, but applying sampling fluids with high osmotic pressure reduces the degree of osmotic shock. Salts used in hank’s balanced salt solution (HBSS) or phosphate-buffered saline (PBS) minimize the osmotic shock. (Brosseau, Vesley, Chen, Gabel, Kuehn and Goyal 1994) In the present study, HBSS without calcium and magnesium was used as impingement fluid. The preparation procedure of HBSS was according to the Sigma-Aldrich (SAFC Biosciences 2006).

### Isolation and amplification of genomic RNA of COVID-19

The viral RNA was extracted from 500 µL of the aerosol specimens using a QIAamp DSP Virus kit (QIAGEN GmbH, Hilden, Germany), according to the manufacturer’s protocols. Then, the quantity and quality of the isolated RNAs were assessed using a NanoDrop™ (Thermo Scientific, Wilmington, USA) spectrophotometer.

The encoding region of the COVID-19 virus envelope (E) and the RNA-dependent RNA polymerase (RdRp) genes were detected using specific primers and probes by real-time polymerase chain reaction (RT-PCR) method (Corman, et al. 2020), with the Rotor-Gene Q (QIAGEN, Germany) instrument as described previously in detail. (Corman, Bleicker, Brünink, Drosten and Zambon 2020) Also, appropriate positive and negative controls were included in each assay.

## Results and discussion

In this study, three zones of sampling were categorized: collected samples from source of SARS-CoV-2 (patient breathing zone), paths of airborne transmission (general area) and receptor of SARS-CoV-2 particles (breathing zone of health care personnel). A total of 20 samples were collected from air to survey the possible occupational exposure of health care personnel with SARS-CoV-2 particles in ambient air of the ICU ward. The results of sample analysis are given in Tables 3, 4 and 5.

**Table 3:** The results of analysis for samples from suspected source of virus release

Sample code	Sample type	RT-PCR test result
A	Source (patient breathing zone)	Positive
H		Positive
G1		Negative
G2		Negative

**Table 4:** The results of analysis for samples from the general area

Sample code	Sample type	RT-PCR test result
C	General area (path of people’s exposure)	Negative
D		Negative
F		Negative
K		Negative
J1		Positive
J2		Negative
J3		Negative
J4		Negative

**Table 5:** The results of analysis for samples from health care personnel breathing zone

Sample code	Sample type	RT-PCR test result
B	Breathing zone	Positive
E	Breathing zone	Negative
G3	Right side of patient bed	Negative
G4	Right side of patient bed	Negative
G5	Left side of patient bed	Negative
G6	Left side of patient bed	Negative
L1	Right side of patient bed	Negative
L2	Left side of patient bed	Negative

As presented in Table 3, the results of RT-PCR analysis confirmed the existence of SARS-CoV-2 in air at two stations of patient breathing zone. All positive samples were related to COVID-19 confirmed patients (samples code: A, and H). The stations with sample codes of G1and G2 belong to COVID-19 suspected patient.

Eight samples belonged to the breathing zone of the health care personnel who were at higher potential risk of infections due to airborne transmission of SARS-CoV-2 through air. (Table 5) All of the samples in this zone were found negative except for B code.

The results of this study showed that airborne emission of COVID-19 through the breathing zone of patients, particularly in ICU wards with confirmed cases of COVID-19, may be higher than in other ICU wards. (Tables 2–3) Almost all patients had general symptoms of SARS-CoV-2 disease (e.g., fever, sneezing, coughing, etc.); thus, the amount of particle release was high. The results of Table 1 show crowded Corona patients (60% of persons in the ICU) and the short term of drug use by patients (maximum 2–4 days) were the main reasons for the high potential of emission of airborne particles due to exhale of patients.

With regards to the virus load emitted by infected individuals, currently no data are available in the scientific literature; therefore, the authors will refer to the case of SARS-CoV-1, which has similar characteristics (Buonanno et al. 2020).

Based on the given results from Table 4, the sampling of general areas from different locations, SARS-CoV-2 may have the potential to be transmitted through aerosols. The result of J1 station proposed this fact, as it was located about 10 m from the ICU unit. The source of the aerosols in this location may be the airborne SARS-CoV-2 from a patient origin directly or may originally result from the direct deposition of respiratory droplets or airborne SARS-CoV-2 from a patient on the personal protective equipment worn by medical staff, professors and resident students and resuspension of virus-laden aerosols from the surface of the protective equipment while they are walking or moving.

Since the health care personnel frequently walk between different wards and stations of ICU, there is a possibility of reaerosolization of SARS-CoV-2 particles. (Table 4).

Therefore, the use of protective strategies and effective control devices is highly recommended.

The final and additional results of the nasopharyngeal swab tests of the suspected patients (cough, fever, sneezing, shortness of breath, etc., with positive CT) were negatively detected and released 2 days after the air sampling tests. Although aerosolization could be considered a third potential route of transmission, along with large droplets emitted from sneezing or coughing and the transmission of viral particles after touching a contaminated surface, the relative contribution of each mode is uncertain. (Ault 2020).

Resuspending particles of a respirable size may be difficult. However, fomites could be transmitted to the hands, mouth, nose or eyes without requiring direct respiration into the lungs. (Council 2020). Thus, in case of lack of adequate protective and control devices or strategies, the suspected COVID-19 patients are faced with the risk of exposure.

Analyzing the breathing zone samples of the health care staff was the most important part of the present study. These results can be used to investigate the relationship between airborne particles and COVID-19, particularly the presence of the virus in the breathing zone of the medical team in the ICU wards. Eight samples were from the breathing zone of the health care personnel who were at higher potential risk of virus contraction due to airborne transmission of SARS-CoV-2. (Table 5) All the samples in this zone were found to be negative except for B code, which might have been due to the accumulation of particles containing inhaled coronavirus caused by 9 confirmed COVID patients at the ICU main hall.

The results of this study highlight the importance of prioritizing the application of control systems to curb aerosol transmission primarily at the source of infection (pollution). In other words, applying control strategies, technologies and devices at the patient zone (especially the breathing zone) will be highly efficient. Also, patient’s bed can be redesigned to allow isolation, venting bed’s air and respiratory treatment of patients.

Also, the results revealed that if health care personnel or patients with suspected symptoms (with negative or unknown PCR result) approach the breathing zone of COVID-19 patients, there would be a high risk of exposure and virus contraction. Also, the health care personnel without proper personal protective equipment (PPE) who are near the breathing zone of COVID-19 patients may be SARS-CoV-2 asymptomatic carriers and contribute to viral transmission through air or even their PPE. The result of airborne sampling at the general area of station J1 proposes this assumption. (Table 4) Also, applying proper and standard ventilating and air-conditioning systems in all parts of indoor spaces at hospitals will be useful and effective in controlling transmission hazard at general areas. In other words, it would limit the path of airborne transmission. One of the main reasons that sampling results at some stations were negative was the use of ventilation systems and pathogenic treatment system in clean areas of the hospital (e.g., health care staff’s rest room) which in turn, resulted in an environment free of airborne particles of SARS-CoV-2. Thus, the necessity of using ventilation systems in closed areas was emphasized. (Janbabai et al. 2020) However, improper design or faults in standard ventilation systems result in a contamination source. (Abouleish 2021) Generally, the positive effect of proper ventilation, operation of heating and air-conditioning systems in reducing SARS-CoV-2 transmission is highlighted by researchers. The relation between improper ventilation systems and outbreak of airborne diseases has been reported previously by WHO. (Chartier and Pessoa-Silva 2009) It was shown that the aerodynamic diameter of particles carrying RNA copies play an important role in aerosolization of virus, and thus, it can be transmitted during talking, sneezing and coughing of a carrier person at distance of 2 m and be viable in air for about 3 h. Fine and very fine particles that remain suspended for hours and travel long distances may transmit SARS-CoV-2 directly if inhaled. (Guzman 2020) Thus, keeping a safe social distance is another strategy to reduce airborne transmission. (Guzman 2020).

## Conclusion

At present, little is known about aerodynamic features and SARS-CoV-2 transmission pathways in aerosols, partly due to problems in sampling virus-containing aerosols in real-world settings and their quantitative challenges in low concentrations.(Liu et al. 2020) The results of this study showed that analysis of gene expression in ambient conditions (indoor air stream) and thereby aerosol transmission of SARS-CoV-2 through air is possible and may lead to occupational exposure of health care personnel. Although there was not confirmation that the infectivity of the viruses detected in this areas of the hospital, it is suggested that SARS-CoV-2 may have the potential to transmit through aerosols. In other words, the aerodynamic size of infectious particles of SARS-CoV-2 was appropriate to remain airborne. (Verreault Moineau and Duchaine 2008) Also, airborne emission of COVID-19 through the breathing zone of patients, particularly in ICU wards with confirmed cases (COVID-19), may be higher than in other ICU wards.

The crowded wards of Corona patients and the short term of drug use by patients can be the main reasons for the high potential of emission (of airborne particles) due to exhale of patients.

The positive result of sample in the suspected patient room (J1 Station) may be related to the frequent movement of professors and resident students from the main ICU hall (with a high pollution load). Thus, in case of lack of adequate protective and control devices or strategies, the suspected COVID-19 patients are faced with the risk of exposure. Understanding the sources and ways of possible exposure are important in applying and selection of protective strategies and effective control technology. No detection of SARS-CoV-2 in most ICU areas, indicating that high rates of air exchange in the intensive care unit, is very effective in limiting SARS-CoV-2 airborne transmission. The results of this study highlight the necessity of applying control systems (as nonthermal plasma and UVGI process) in emission source (patient’s bed) to prevent the exposure risk of the health care personnel and others in the ICU Wards of hospital.
